# Flow behavior of N_2_ huff and puff process for enhanced oil recovery in tight oil reservoirs

**DOI:** 10.1038/s41598-017-15913-5

**Published:** 2017-11-16

**Authors:** Teng Lu, Zhaomin Li, Jian Li, Dawei Hou, Dingyong Zhang

**Affiliations:** 1 School of Petroleum Engineering, China University of Petroleum, Qingdao, 266580 China; 2 Sinopec Shengli Oilfield Company, Dongying, 257000 China

## Abstract

In the present work, the potential of N_2_ huff and puff process to enhance the recovery of tight oil reservoir was evaluated. N_2_ huff and puff experiments were performed in micromodels and cores to investigate the flow behaviors of different cycles. The results showed that, in the first cycle, N_2_ was dispersed in the oil, forming the foamy oil flow. In the second cycle, the dispersed gas bubbles gradually coalesced into the continuous gas phase. In the third cycle, N_2_ was produced in the form of continuous gas phase. The results from the coreflood tests showed that, the primary recovery was only 5.32%, while the recoveries for the three N_2_ huff and puff cycles were 15.1%, 8.53% and 3.22%, respectively.The recovery and the pressure gradient in the first cycle were high. With the increase of huff and puff cycles, and the oil recovery and the pressure gradient rapidly decreased. The oil recovery of N_2_ huff and puff has been found to increase as the N_2_ injection pressure and the soaking time increased. These results showed that, the properly designed and controlled N_2_ huff and puff process can lead to enhanced recovery of tight oil reservoirs.

## Introduction

The soaring global energy demand coupled with the declining conventional oil production has resulted in an increased emphasis on harnessing unconventional resources, such as the tight oil. However, the primary recovery is between 5% and 10% of the original oil in place (OOIP) in tight oil reservoirs, even after long horizontal wells have been drilled and massively fractured. The performance of conventional waterflooding to recover tight oil reservoirs is bad mainly due to the extremely low injectivity^1–5^.

Recently, there have been studies focusing on CO_2_ huff and puff in tight oil reservoirs. The CO_2_ huff and puff process is a typical single well operation, usually involving three steps, which are injecting a pre-determined slug of CO_2_, giving the soaking time to allow the gas phase to mix with the oil phase in place, and producing the oil immediately after the soaking operation. Yu *et al*.^6^ used numerical reservoir simulations to model the CO_2_ injection as a huff and puff process based upon typical reservoir and fracture properties taken from the Bakken formation. Sanchez-Rivera *et al*.^7^ used a compositional reservoir simulator (CMG GEM) to study different design components of the huff and puff process to identify the parameters having the largest impact on the recovery of oil, and to understand the reservoir’s response to cyclical gas injection. Yang *et al*.^8^ investigated the fluid injectivity and oil recovery of water-alternating-CO_2_ processes in tight oil formations. Ma *et al*.^9^ studied the viability of CO_2_ huff and puff process as the primary means to enhance oil recovery in low-pressure tight reservoirs, and thereby optimized the corresponding key operating parameters of the process. Besides, it is of fundamental and practical importance to study the interfacial tension (IFT) between the crude oil and the gas at high pressures and elevated temperatures. Gu *et al*.^10^ measured the CO_2_ solubilities in a crude oil and the equilibrium IFTs of the crude oil-CO_2_ system under different equilibrium pressures and at 27 °C. Ayatollahi *et al*.^11^ investigated the IFT to assess the impact of temperature, pressure, and paraffin type on a N_2_ injection process as an efficient enhanced oil recovery method.

In recent years, many scholars have conducted studies on the development of tight oil reservoirs with CO_2_ injection. However, studies focusing on N_2_ injection are relatively scarce in literature. N_2_ is relatively difficult to dissolve in crude oil, and has higher miscible pressure than CO_2_. Therefore, the effect of N_2_ injection is generally not as good as that of the CO_2_. As for some tight oil fields in China, the feasibility of N_2_ injection is high, mainly for the following reasons. 1) The sources of CO_2_ are limited, whereas the cost of CO_2_ injection is high. Furthermore, N_2_ can be separated from air, and the cost of this process is relatively low^12^. 2) CO_2_ has strong corrosive characteristics, and can severely corrode the tubular columns and pipe networks. Corrosion protection is not considered in many oil fields, and therefore, the risk posed by CO_2_ injection is very high. 3) CO_2_ may cause asphaltene deposition, while the deposited asphaltene can block the tight oil reservoir. Since the pore of tight oil itself is very tiny, the blocking will damage the formation^13^. Therefore, in the present study, the performance of N_2_ huff and puff process has been investigated in tight oil reservoirs. The objective of this paper was to evaluate the potential of N_2_ huff and puff process to enhance the recovery of tight oil reservoirs. The interfacial tension (IFT) between the N_2_ and the crude oil was measured at different pressures. N_2_ huff and puff tests were performed in micromodels and tight oil cores to investigate the microscopic flow behaviors of different huff and puff cycles, and the effect of various operating parameters on oil recovery.

## Experimental

### Materials

NaCl solution with the salinity of 3500 mg/L was used as the injected brine. The crude oil used in the experiments was collected from Shengli Oilfield, whereas its properties are shown in Table 1. The results from the compositional analysis of the light crude oil were obtained using the standard ASTM D86, and are presented in Table 2.

**Table 1:** Properties of the crude oil.

Oil Density (kg/m^3^) 50 °C	Oil viscosity (mPa·s) 50 °C	Saturated (wt%)	Aromatic (wt%)	Resin (wt%)	Asphaltenes (wt%)
848.7	15.9	55.94	37.28	3.25	3.53

**Table 2:** Compositional analysis of the crude oil.

Carbon no.	mol.%	Carbon no.	mol.%	Carbon no.	mol.%
C_1_	0	C_18_	2.95	C_35_	0.55
C_2_	0	C_19_	2.35	C_36_	0.39
C_3_	0.1	C_20_	2.04	C_37_	0.32
C_4_	1.12	C_21_	1.95	C_38_	0.31
C_5_	2.57	C_22_	1.68	C_39_	0.29
C_6_	4.68	C_23_	1.62	C_40_	0.27
C_7_	10.34	C_24_	1.38	C_41_	0.25
C_8_	6.58	C_25_	1.65	C_42_	0.21
C_9_	8.24	C_26_	1.57	C_43_	0.19
C_10_	6.21	C_27_	1.32	C_44_	0.17
C_11_	3.24	C_28_	1.05	C_45_	0.15
C_12_	5.64	C_29_	0.98	C_46_	0.14
C_13_	5.21	C_30_	0.65	C_47_	0.13
C_14_	2.54	C_31_	0.95	C_48_	0.13
C_15_	3.57	C_32_	0.87	C_49_	0.12
C_16_	3.68	C_33_	0.58	C_50+_	5.34
C_17_	3.21	C_34_	0.52	Total	100

**Figure 1:**
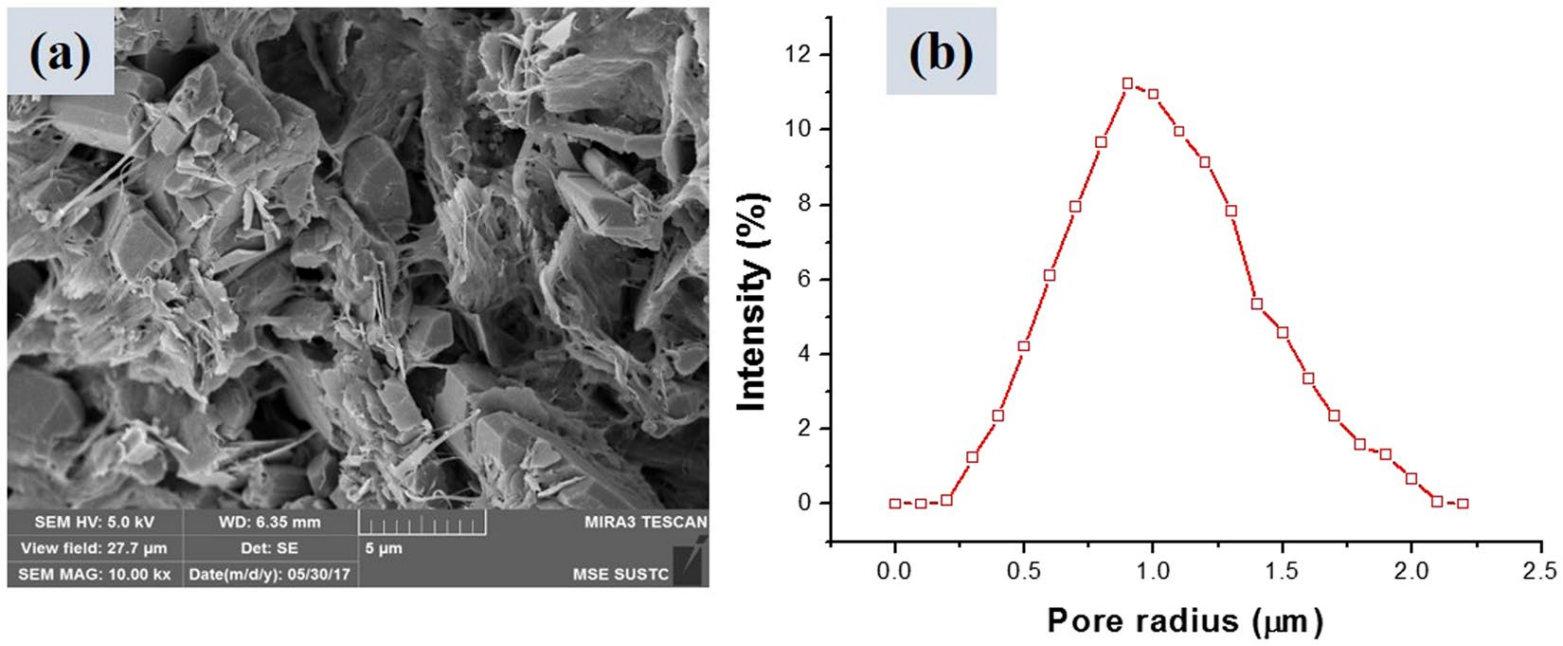
(**a)** SEM of the tight oil core. **(b)** Distribution of the core radius for the tight oil core.

The tight oil cores used in this study were of cylindrical shape, had the lengths and diameter of 10 cm and 2.5 cm, respectively, and were extracted from the Shengli Oilfield. The core samples were washed using various solvents (Soxhlet extraction with xylene, methanol, and chloroform) prior to further use. The morphology of grains and pores in the core sample was studied using the scanning electron microscope (SEM), as shown in Fig. 1(a). Mercury intrusion capillary pressure (MICP) was used to determine the pore size distribution. The measurements were conducted on a small piece of low permeability core, which represented an off-cut from the cores used in the coreflood tests. Figure 1(b) shows the distribution of pore radius for the tight oil core. The mineralogical composition of the cores was analyzed using X-Ray diffraction (XRD), and the results are given in Table 3.

**Table 3:** Mineralogy of low permeability cores.

Mineral	Concentration (wt%)
Quartz	38.8
Feldspar	35.9
Debris	17.2
Kaolinite	3.6
Chlorite	1.2
Others	3.3

## Methods

### Measurement of the interfacial tension (IFT)

**Figure 2:**
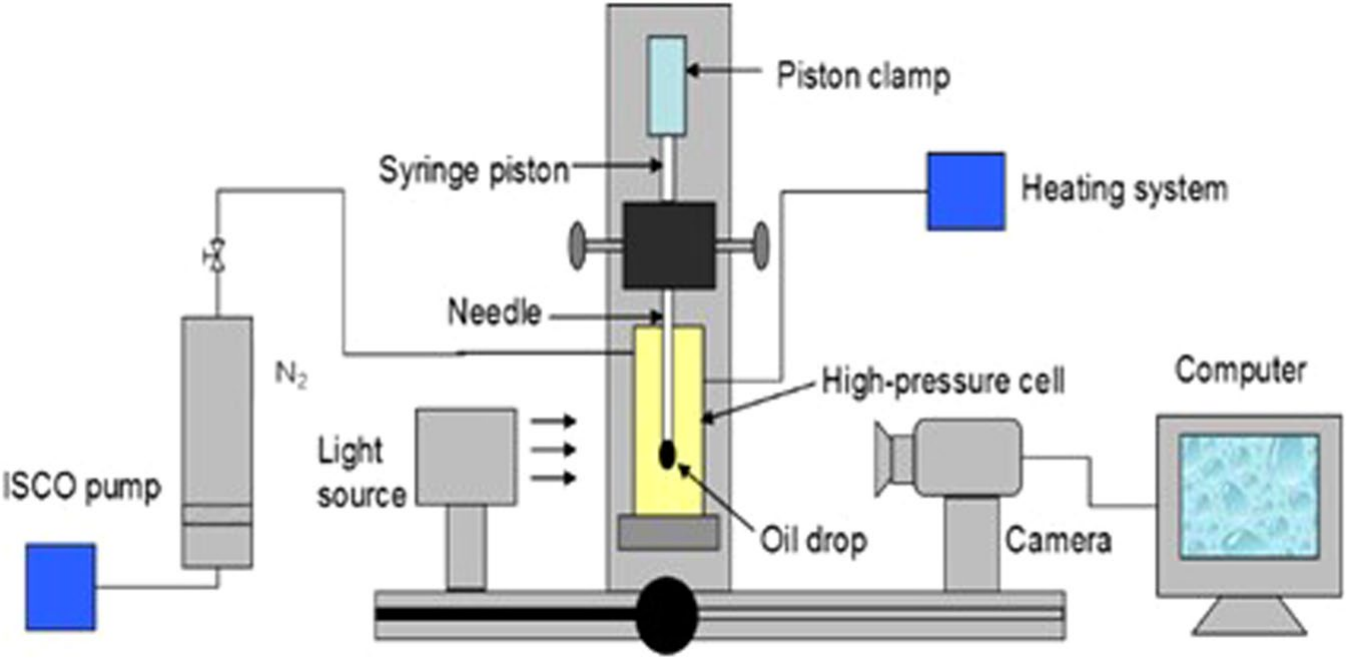
Schematic of the pendant drop tensiometer.

The study of IFT between the crude oil and the N_2_ at high pressures is of significance. To investigate the effectiveness of pressure in reducing the crude oil/N_2_ IFT, the IFT behavior of crude oil/N_2_ system was studied at different pressures. The IFT between the crude oil and the N_2_ at different pressures was measured using the commercial pendant drop tensiometer (Tracker, Teclis), as shown in Fig. 2. In the center of the experimental setup, a high-pressure cell (with the see-through window) was connected to a N_2_ cylinder. The pressure of N_2_ was controlled using the ISCO pump. The experimental temperature was maintained using the heating system. Based upon the force balance between the Laplace and the head pressure on the drop, the computer analyzed images of the oil drop shape were solved for the IFT values.

The IFT values of the crude oil-N_2_ system were measured at 0.5–20.5 MPa and 60 °C. Prior to each experiment, the high-pressure cell was thoroughly cleaned with ethanol, and then flushed with N_2_. The pressure cell was filled with N_2_ to the required pressure and temperature. After the pressure and temperature inside the pressure cell stabilized, the oil was introduced to the pressure cell from the sample cylinder to form a pendant oil drop at the tip of syringe needle. Once a well-shaped pendant drop was formed, the sequential digital images of the dynamic pendant oil drop at different time intervals were acquired and stored automatically in the computer. Thereafter, the dynamic and equilibrium IFTs were determined by solving the Laplace equation of capillarity. Each IFT measurement was repeated three times to ensure satisfactory repeatability for a certain condition of pressure and temperature.

### Microscopic visual experiments

**Figure 3:**
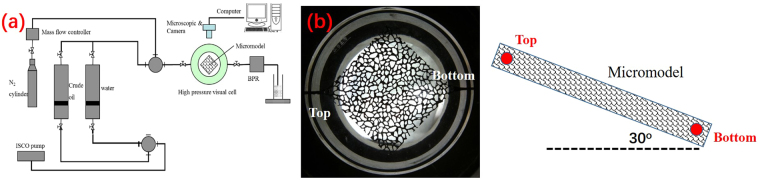
(**a**) Schematic of the microscopic visual experimental setup. (**b**) Image of the micromodel.

The microscopic visual experiment was used to investigate the flow behaviors of N_2_ and oil during the N_2_ huff and puff process. The schematic of the microscopic visual experiment is shown in Fig. 3a. A quarter 5-spot glass-etched micromodel was installed in the high pressure visual cell under high pressure, and was placed at the dip angle of 30° with the level line, as shown in Fig. 3b. The high-pressure visual cell allowed for an overburden pressure of up to 15 MPa and a temperature of up to 120 °C. The confinement fluid used in the visual cell was water.

The experimental procedure used was as follows. 1) The micromodel was vacuumed using the vacuum pump. 2) After being vacuumed, the micromodel was saturated with brine. 3) The micromodel was displaced by crude oil with the back pressure of 12 MPa until water egress ceased. 4) To simulate the primary production, the outlet at the bottom of micromodel was opened, and the pressure in the micromodel was gradually reduced. 5) After the primary production, N_2_ was injected into the micromodel from the outlet at the bottom until the micromodel pressure reached 12 MPa. 6) The micromodel was soaked for 24 hours. The injected N_2_ could flow to the top of micromodel. 7) The outlet at the bottom of micromodel was opened to gradually reduce the back pressure until the micromodel pressure reached zero. 8) N_2_ was injected again, and then, the next cycle of N_2_ huff and puff process was carried out. The experimental temperature was maintained at 60 °C. During the experiment, a digital camera (Model L110; Nikon) was used to record the images within the micromodel.

### Coreflood experiments

**Figure 4:**
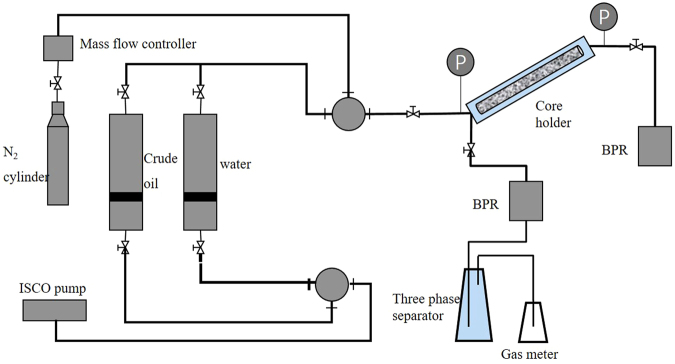
Schematic of the coreflood test.

Figure 4 shows the schematic of the setup used to conduct coreflood experiments. The tight oil core was placed in the core holder, which can be set at a certain dip angle according to the requirement. The experimental temperature was maintained at 60 °C. The experimental procedure used was as follows. 1) The tight oil core was vacuumed and saturated with brine, while the porosity of the core was obtained. 2) At the back pressure of 20 MPa, 10 pore volume (PV) of brine was injected into the core at the injection rate of 0.01 ml/min. The core permeability was measured using the Darcy law. 3) The initial oil saturation and the connate water saturation across the core were established by injecting crude oil into the core until no additional water came out. 4) After the core was saturated with crude oil, the top outlet of the core holder was closed, while the bottom outlet of the core holder was opened. The back pressure was gradually reduced to simulate the primary production. 5) After the primary production, N_2_ was injected into the core to a certain pressure through the bottom of core holder. Then, the core was soaked. The purpose of soaking process is to allow the injected N_2_ to flow into the deeper of the core. 6) The bottom outlet of the core holder was opened to gradually reduce the back pressure. 7) After the pressure was reduced to zero, N_2_ was reinjected and then, the next cycle of N_2_ huff and puff process was conducted.

## Results and Discussion

### IFT measurements

**Figure 5:**
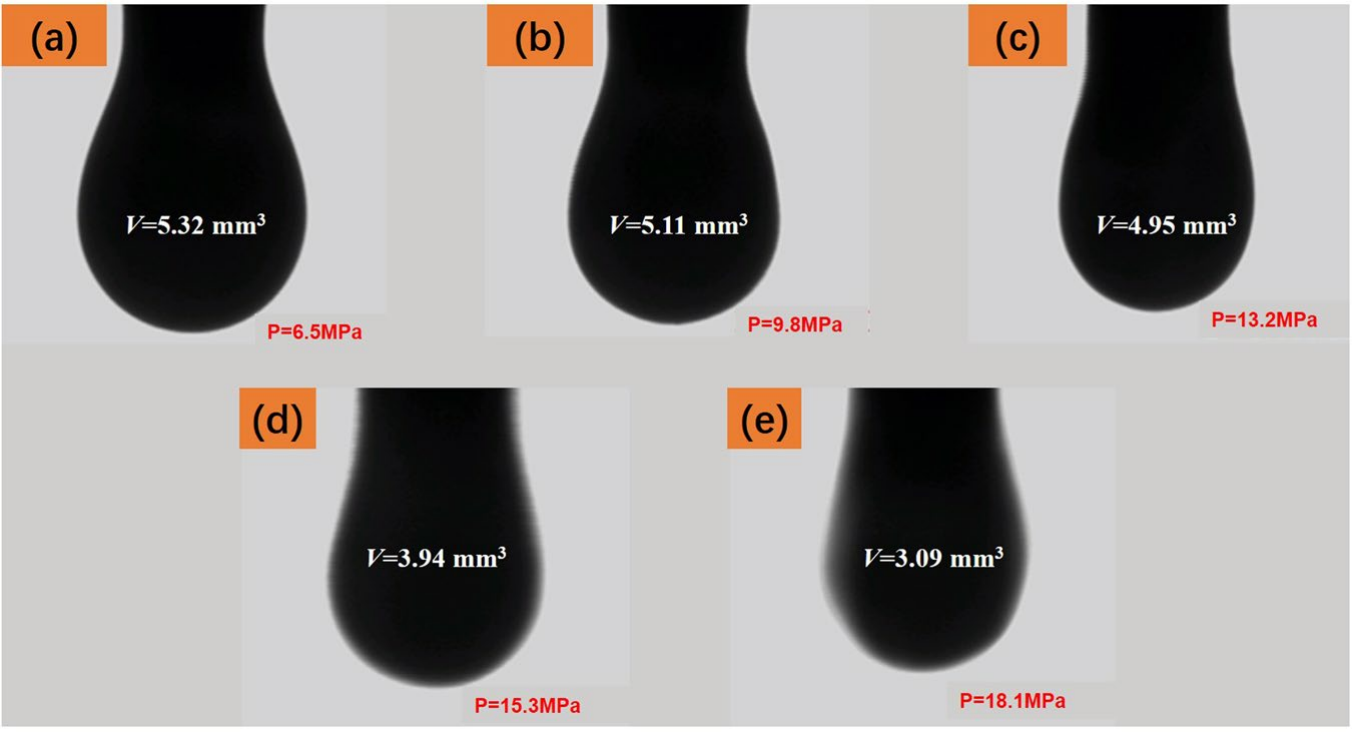
Digital images of the pendant drops surrounded by N_2_ at different pressures and 60 °C.

The IFT between the crude oil and the N_2_ was measured at 0.5–20.5 MPa and 60 °C. The sequential digital images of the dynamic pendant oil drops and their volumes at different pressures are shown in Fig. 5. As shown in Fig. 5(a–c), the volume of oil drop decreased slightly with pressure until the pressure reached *ca*. 14 MPa. For pressure higher than *ca*. 14 MPa (Fig. 5(d,e)), the volume of oil drop decreased rapidly with pressure, whereas the oil–N_2_ interface also became blurred.

**Figure 6:**
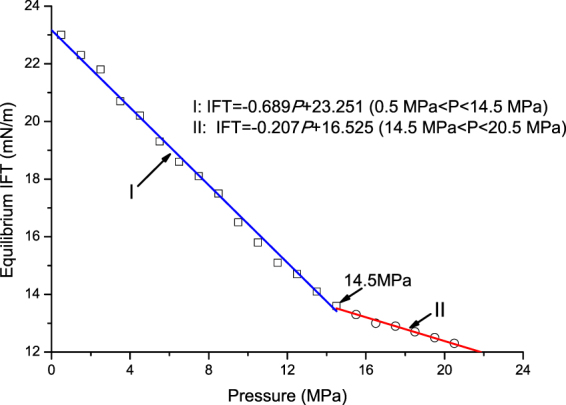
IFTs between the oil sample and the N_2_ at different pressures.

Figure 6 shows the IFTs between the crude oil and the N_2_ at different pressures. It is observed that the IFT decreased almost linearly with pressure, and was divided into two pressure ranges. The first pressure range was 0.5–14.5 MPa, while the second was 14.5–20.5 MPa. Based on the experimental data shown in Fig. 6, the equilibrium IFT was linearly fitted with the pressure in both the pressure ranges. The results for regression analysis are summarized in Table 3. Because more N_2_ will dissolve in oil at higher pressure, the IFT linearly decreased with pressure. As shown in Fig. 6 and Table 3, the rate of decrease of IFT in the higher-pressure range was less than that in the lower-pressure range. It is well known that the asphaltenes molecules disperse in oil, and form a relatively homogeneous solution at low pressures. As the pressure increases, the concentration of N_2_ increases, due to which, the asphaltenes molecules flocculate due to the inverse relationship between the pressure and the oil composition^14,15^. The interfacial activity of colloidal solutions usually increases due to the tendency of particles to accumulate at the interfaces. This accumulation of particles can also occur at the oil–N_2_ interface, which leads to the changes in IFT values. Therefore, it is concluded that large molecules of asphaltenes began to aggregate at the N_2_-oil interface, which caused the IFT to slightly increase in the high-pressure range. Due to the asphaltenes’ agglomeration, the asphaltenes’ film was formed at the oil–N_2_ interface and modified the IFT - pressure trend due to the effect of N_2_ solubility, as shown in Fig. 6. In other words, the asphaltenes’ film formed at the oil–N_2_ interface in the high-pressure range reduced the N_2_ dissolution in the oil. As a result, the rate of decrease of IFT in the higher-pressure range was lower than that in the lower-pressure range. Therefore, when the pressure was higher than 14.5 MPa, the volume of oil drop decreased rapidly with pressure, while the oil–N_2_ interface became blurred.

### Microscopic visual experiments

**Figure 7:**
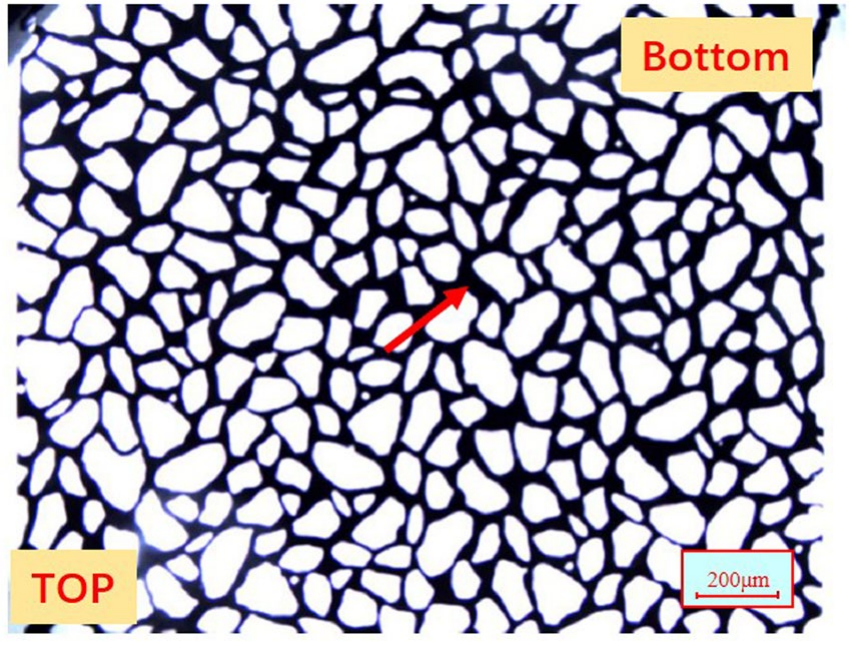
Microscopic image of the primary production.

To investigate the flow characteristics during the N_2_ huff and puff process, the microscopic visual experiments were carried out. Firstly, the primary production was conducted in the micromodel. The micromodel pressure was reduced from 12 MPa to zero with the pressure drop rate of 1 MPa/h. Video Clip 1 (see the Supporting Information) and Fig. 7 depict the video and image of primary production in the micromodel. It can be observed that no N_2_ was produced in the micromodel, and only the oil phase flow existed in the process.

**Figure 8:**
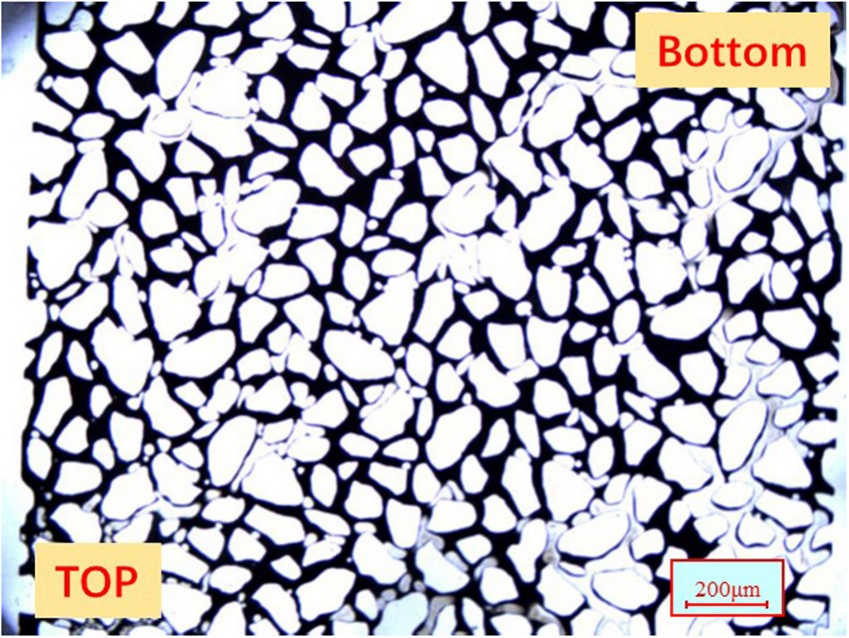
Microscopic image of the first N_2_ huff and puff cycle.

After the primary production, the N_2_ huff and puff experiment was carried out. N_2_ was injected into the micromodel until the micromodel pressure reached 12 MPa. After the micromodel was soaked for 24 hours, the pressure in the micromodel was gradually reduced from 12 MPa to zero with the rate of pressure drop being 1 MPa/h. Video Clip 2 (see the Supporting Information) and Fig. 8 present the results of the first cycle of N_2_ huff and puff process. It can be seen that, during the first cycle of N_2_ huff and puff process, the N_2_ was produced with crude oil from the bottom outlet of the micromodel. The produced N_2_ was dispersed in crude oil in the form of bubbles, forming the foamy oil flow. The foamy oil is often used to describe a discontinuous gas phase dispersed in the continuous oil phase^16,17^. The results of microscopic visual experiments showed that the foamy oil flow was observed during the first cycle of N_2_ huff and puff process for the light oil.

**Figure 9:**
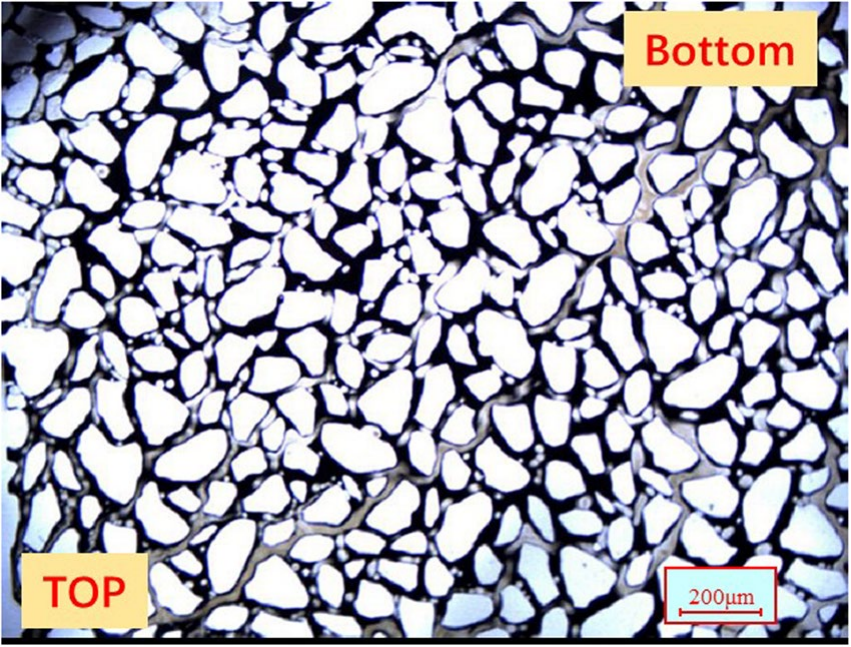
Microscopic image of the second N_2_ huff and puff cycle.

After the first cycle of N_2_ huff and puff process, the second cycle was conducted in the micromodel. Video Clip 3 (see the Supporting Information) and Fig. 9 present the results of the second cycle of N_2_ huff and puff process. It can be seen that, the microscopic flow characteristics in the second cycle were different from those in the first cycle. The gas saturation in the micromodel obviously increased. The N_2_ was produced not only in the form of gas bubbles, but also in the form of continuous gas phase. The continuous gas phase meant that the gas bubbles were not dispersed in the oil phase, but coalesced to be continuous. Due to the formation of continuous phase, the gas channel was observed in the second cycle.

**Figure 10:**
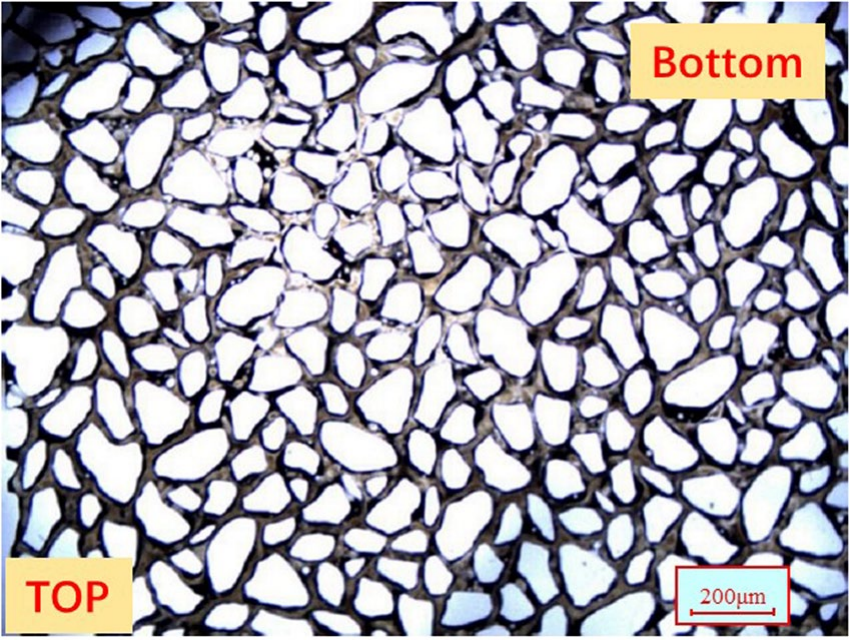
Microscopic image of the third N_2_ huff and puff cycle.

The third cycle of N_2_ huff and puff was conducted at the end of the second cycle. Video Clip 4 (see the Supporting Information) and Fig. 10 present the video and images of the third cycle of N_2_ huff and puff process. It can be seen that, in the third cycle of N_2_ huff and puff process, the N_2_ was produced not only in the form of gas bubbles, but also in the form of continuous phase. The foamy oil flow was not observed in the third cycle. However, the gas channel problem was found to be very severe in the third cycle.

Based on the microscope visual experiments, it is found that the flow behaviors of N_2_ were different in each cycle of N_2_ huff and puff process. In the first cycle of N_2_ huff and puff process, the N_2_ was mainly dispersed in the crude oil in the form of bubbles, thereby forming the foamy oil flow. In the second cycle, the number of bubbles reduced, while the continuous gas phase was observed. This means that the characteristics of the foamy oil flow were weakened in the second cycle. In the third cycle of N_2_ huff and puff process, N_2_ was produced in the form of continuous gas phase, while the foamy oil flow was not observed.

### Coreflood experiments

To study the potential of N_2_ huff and puff process for enhanced tight oil recovery, five coreflood experiments were carried out. In each experiment, three huff and puff cycles were conducted. The effects of N_2_ injection pressure, soaking time and dip angle on the N_2_ huff and puff process were analyzed. The experimental parameters and the results are shown in Table 4.

**Table 4:** Experimental parameters and results of coreflood experiments.

Test #	Permeability (md)	Porosity (%)	Initial oil saturation (%)	Cycle number	N_2_ injection pressure (MPa)	Soaking Time (h)	Dip angle (degree)	Oil recovery (%)
1	0.089	8.95	65.27	1st	20	24	30	15.1
				2nd	20	24	30	8.53
				3rd	20	24	30	3.22
2	0.081	8.22	64.13	1st	15	24	30	13.21
				2nd	15	24	30	7.25
				3rd	15	24	30	3.32
3	0.093	8.35	66.17	1st	10	24	30	9.35
				2nd	10	24	30	6.32
				3rd	10	24	30	3.36
4	0.086	8.68	64.39	1st	20	12	30	12.5
				2nd	20	12	30	7.14
				3rd	20	12	30	3.01
5	0.092	8.97	67.14	1st	20	12	0	12.14
				2nd	20	12	0	6.35
				3rd	20	12	0	2.95

#### Performance of the N_2_ huff and puff process in different cycles

**Figure 11:**
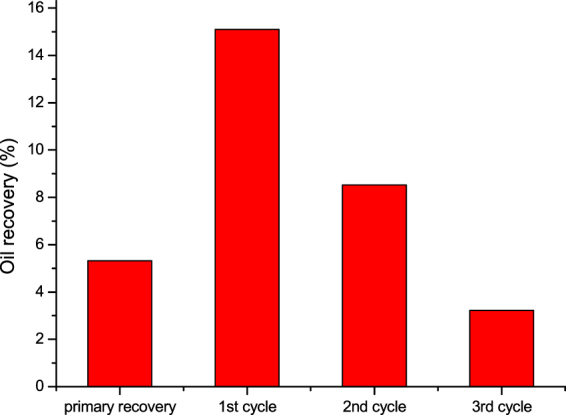
Oil recovery of the primary production and the three cycles of N_2_ huff and puff.

In Run 1, the N_2_ injection pressure was set to be 20 MPa, while the soaking time was 24 h. Additionally, the core dip angle was set to be 30° in the three cycles of huff and puff process. Figure 11 shows the oil recovery of the primary production and the three cycles of N_2_ huff and puff process. It can be seen that the primary recovery was only 5.32%. The recovery in the first cycle of N_2_ huff and puff process was 15.1%, while the recoveries in second and third cycles were 8.53% and 3.22%, respectively. This means that the recovery performance of the first cycle was good. With the increase in the huff and puff cycles, the recovery decreased rapidly.

**Figure 12:**
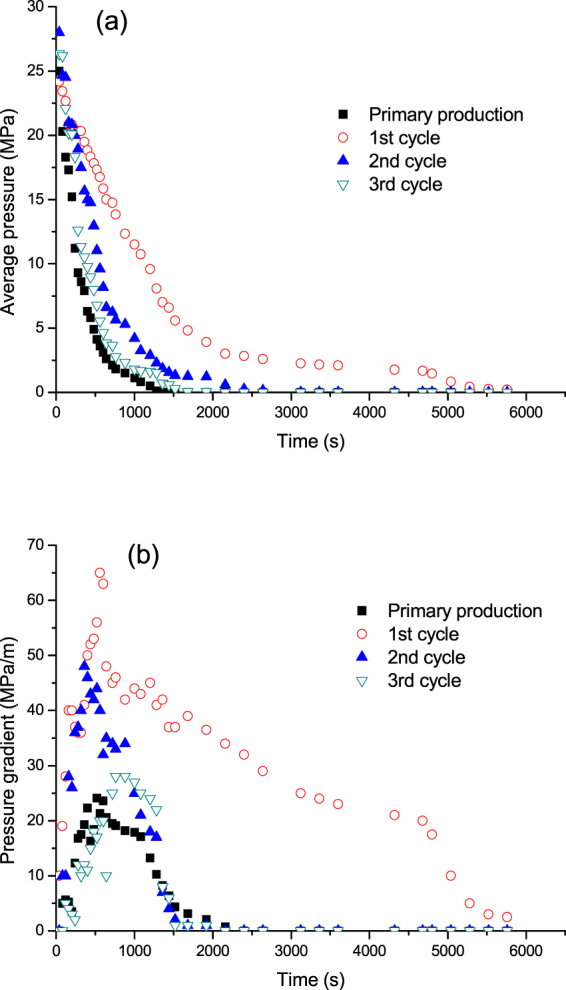
Average pressure and the pressure gradient during the primary production and three cycles of N_2_ huff and puff cycles. (**a**) Average pressure. (**b**) Pressure gradient.

Figure 12 shows the average pressure and the pressure gradient in the cores during the primary production and the three cycles of N_2_ huff and puff process. It can be seen that, in the primary production stage, the average pressure of the core dropped quickly while the pressure gradient was low. This was due to the oil phase flow in the primary production (Video Clip 1). The transient pressure disturbance was due to the oil production at the outlet. During the first cycle of N_2_ huff and puff process, the average pressure dropped slowly, while the pressure gradient was much higher than that in the primary production. However, with the increase in huff and puff cycles, the core pressure decreased rapidly, while the pressure gradient became low, especially in the third cycle of huff and puff process. This was because, in the first cycle of N_2_ huff and puff process, the foamy oil was formed. The bubbles were able to increase the elastic energy of the fluid, which can increase the pressure gradient and the average pressure in the core. However, with the increase in huff and puff cycles, the foamy oil flow characteristic weakened, and more N_2_ was produced in the form of a continuous gas phase. Therefore, the pressure gradient became low, while the pressure dropped rapidly.

**Figure 13:**
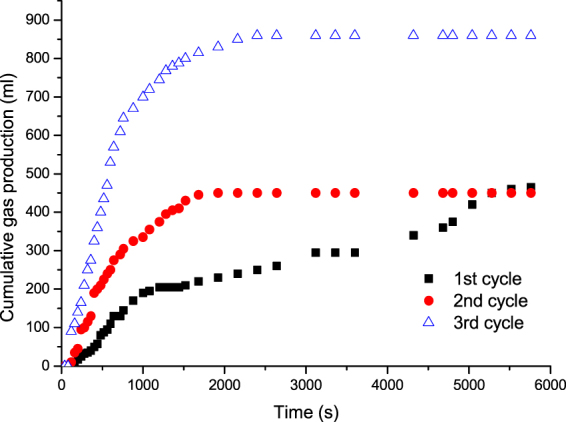
Cumulative N_2_ production in different huff and puff cycles.

Figure 13 shows the cumulative N_2_ production during the three cycles of N_2_ huff and puff process. It can be observed that, in the first cycle, the N_2_ production rate and the cumulative N_2_ production were low due to the formation of foamy oil flow. The N_2_ was produced in the form of gas bubbles, while it did not form a continuous gas phase. It can be concluded that the foamy oil flow can keep more N_2_ stored in the tight cores to increase the elastic energy to drive the oil. With the increase in huff and puff cycles, the continuous gas phase gradually formed. As a result, the N_2_ production rate and the cumulative N_2_ production increased. The gas channels were formed, due to which, little oil can be recovered because of the viscous fingering caused by the adverse mobility ratio between the oil and the gas.

**Figure 14:**
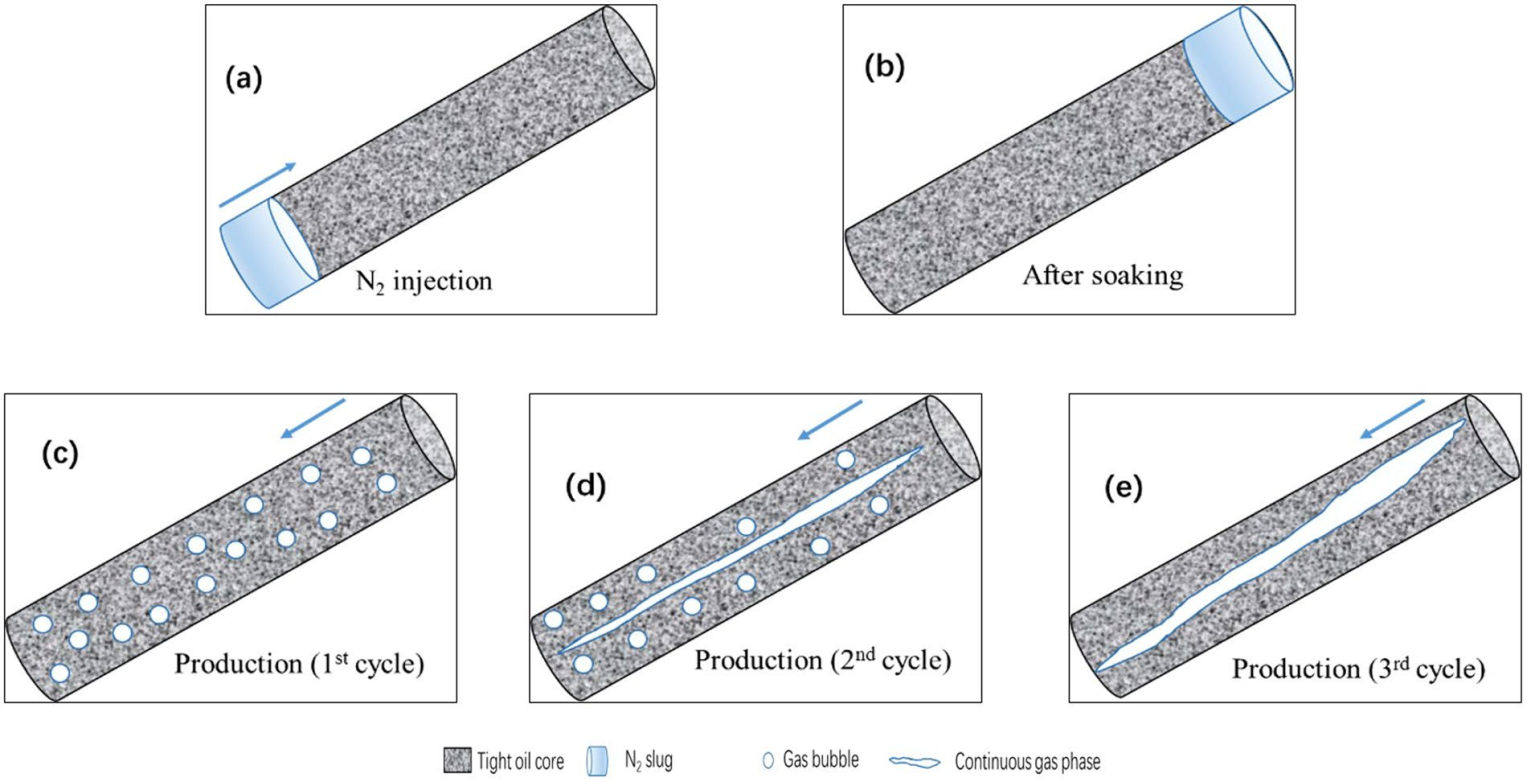
Schematics of the flow behaviors in N_2_ huff and puff process. (**a**) N_2_ was injected from the bottom of core. (**b**) N_2_ migrated to the top of core after the soaking time. (**c**) First huff and puff cycle. (**d**) Second huff and puff cycle. (**e**) Third huff and puff cycle.

Based on the microscopic visual and the coreflood experiments, the flow behaviors of N_2_ huff and puff process could be concluded, as shown in Fig. 14. Figure 14a shows the injection of N_2_ from the bottom of tight oil core. The injected N_2_ could migrate to the top of core under gravity differentiation after the soaking time (Fig. 14b). During the production period of the first cycle, N_2_ was produced with oil in the form of foamy oil flow, as shown in Fig. 14c. The foamy oil was able to increase the elastic energy and pressure gradient of cores, thereby improving the recovery performance of the first cycle of N_2_ huff and puff process. Then, in the second cycle of N_2_ huff and puff process, the foamy oil flow weakened, while N_2_ gradually accumulated in the continuous gas phase, as shown in Fig. 14d. Therefore, the pressure gradient in the core decreased, while the N_2_ production rate increased. The oil recovery of the second cycle was less than that of the first cycle. In the third cycle of N_2_ huff and puff process, N_2_ was produced mainly in the form of continuous gas phase (Fig. 14e), due to which, the gas channeling was severe in the third cycle. Therefore, the recovery performance of the third cycle was poor.

#### Effect of N_2_ injection pressure

**Figure 15:**
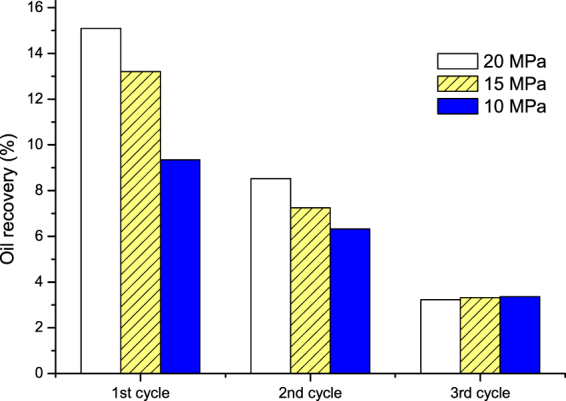
Oil recovery of N_2_ huff and puff process under different injection pressures.

**Figure 16:**
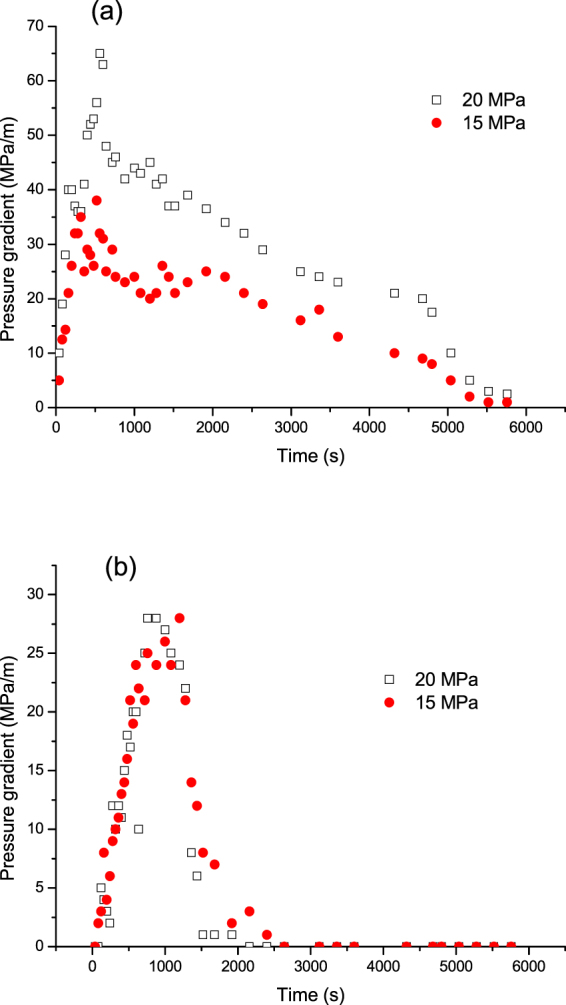
Pressure gradient during the first and third cycles of N_2_ huff and puff process at different injection pressures. (**a**) First cycle. (**b**) Third cycle.

Figure 15 shows the oil recovery of the three (1–3) cycles of N_2_ huff and puff process under different N_2_ injection pressures. The soaking time was 24 hours, whereas core dip angle was set at 30°. It can be seen that, for a higher N_2_ injection pressure, the oil recoveries in the first and second cycles were higher. The injection pressure showed little effect on the recovery of oil in the third cycle. Figure 16 shows the pressure gradient during the first and third cycles of N_2_ huff and puff process at the injection pressures of 20 and 15 MPa. It can be seen in Fig. 16a that, in the first cycle, the pressure gradient for 20 MPa was obviously higher than that for 15 MPa. It means that, increasing the N_2_ injection pressure effectively enhanced the elastic energy of the tight core in the first cycle of N_2_ huff and puff process, thereby improving the recovery performance in the first cycle. However, as shown in Fig. 16b, in the third cycle of N_2_ huff and puff process, the pressure gradients for 20 and 10 MPa pressures were similar.

**Figure 17:**
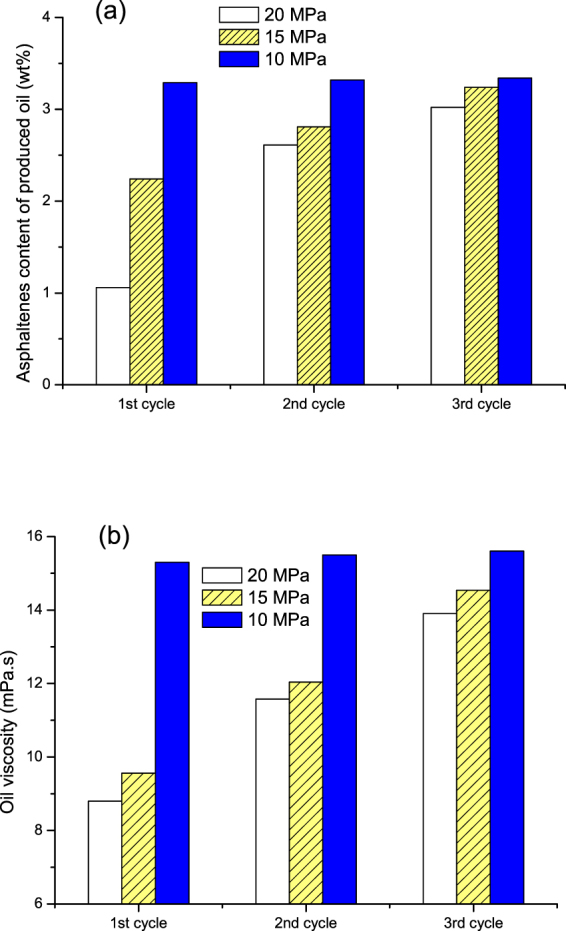
Asphaltenes content and the viscosity of produced oil in the N_2_ huff and puff process at different injection pressures.

Figure 17 shows the asphaltenes content and the viscosity (at 50 °C) of the produced oil in the primary production and the three cycles of N_2_ huff and puff process at different pressures. It can be seen that the contents of asphaltenes and the viscosities of produced oil at 20 and 15 MPa were much lower than that at 10 MPa. The contents of asphaltenes in the produced oil at 10 MPa were close to the corresponding value in the original crude oil (3.53%). Based upon the IFT results, the asphaltenes’ precipitation amount was small for pressures less than 14.5 MPa, as shown in Fig. 5. As the pressure became higher than 14.5 MPa, a large amount of asphaltenes precipitated in the porous media. The asphaltenes could adsorb onto the sand grains, and gather in the pore, thereby reducing the content of asphaltenes and the viscosity of the produced oil.

#### Effect of soaking time

**Figure 18:**
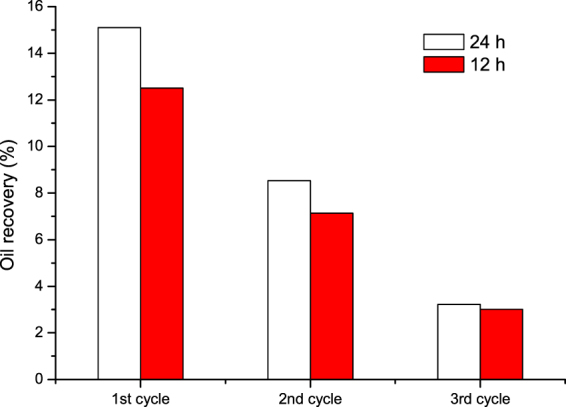
Oil recovery of N_2_ huff and puff process for different soaking times.

**Figure 19:**
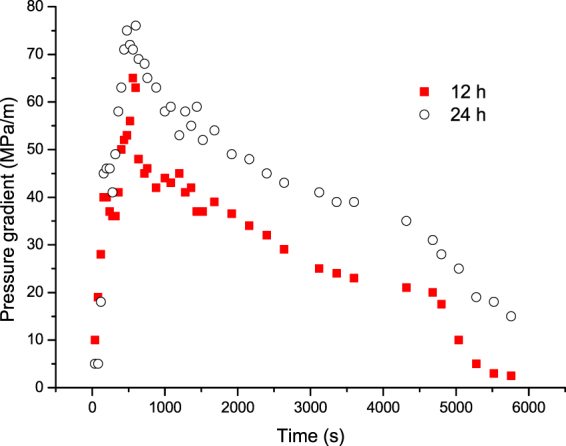
Pressure gradient of the first cycle of N_2_ huff and puff process for different soaking times.

**Figure 20:**
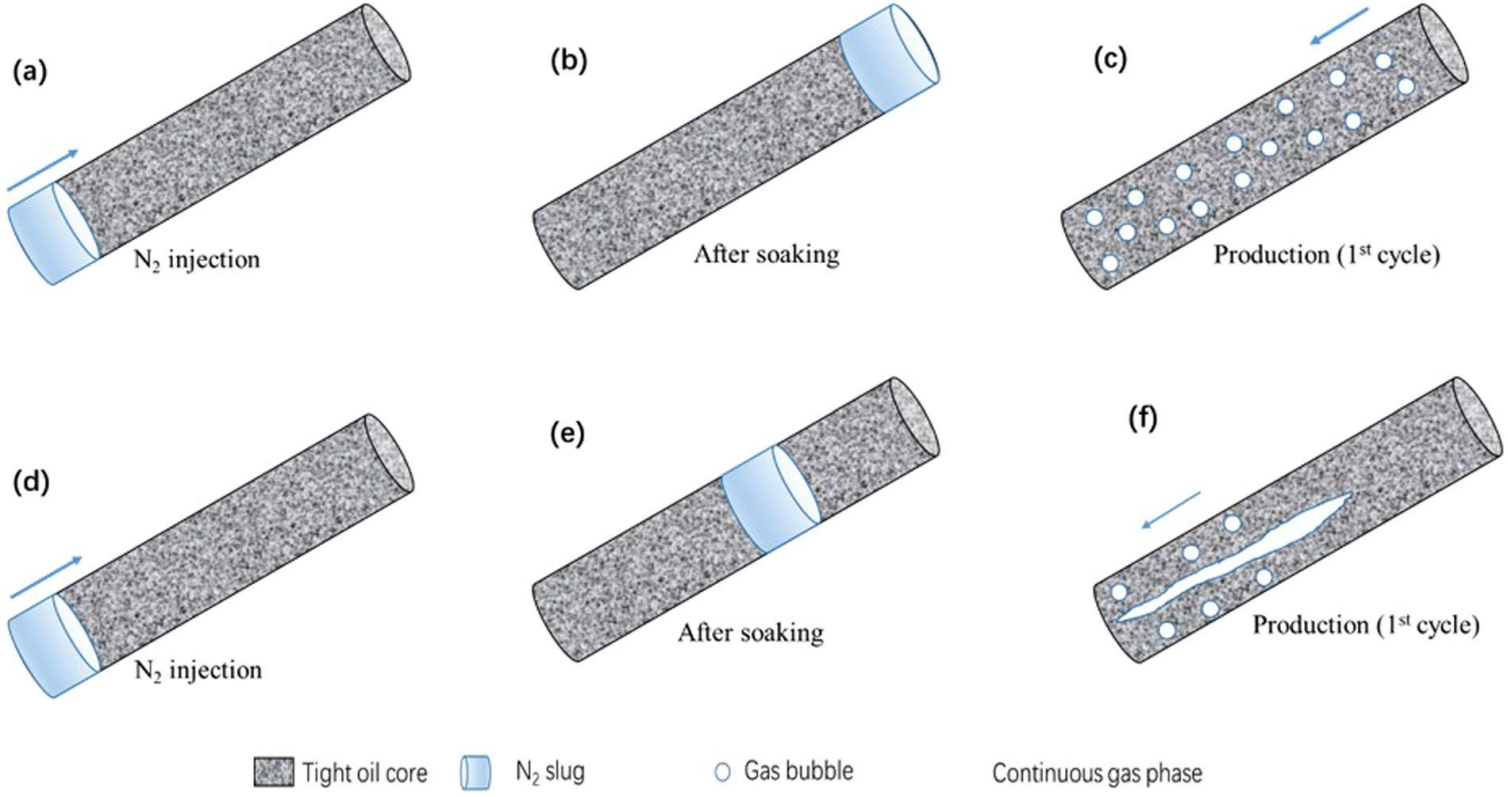
Schematics of the N_2_ huff and puff process for different soaking times. (**a**–**c**) Soaking time of 24 h. (**d**–**f**) Soaking time of 12 h.

Figure 18 compares the oil recovery for the three (1–3) cycles of N_2_ huff and puff process for the soaking times of 12 h and 24 h. It can be seen that the oil recovery for the soaking time of 24 h was higher than that for the soaking time of 12 h. Figure 19 shows the pressure gradient of the first cycle of N_2_ huff and puff process for different soaking times. It is observed that the pressure gradient for the soaking time of 24 h was obviously higher than that for the soaking time of 12 h. To illustrate the mechanism, the schematic diagrams of N_2_ huff and puff process for different soaking times are shown in Fig. 20. Figure 20(a–c) show the schematic diagrams for the soaking time of 24 h, while Fig. 20(d–f) show the schematic diagrams for the soaking time of 12 h. The injected N_2_ could flow into the higher region of the core for the soaking time of 24 h, while the N_2_ slug was far away from the outlet at the bottom of the core (Fig. 20b). In the production period, N_2_ was easily sheared into the bubbles by sand grains, due to which, the foamy oil flow became more stable (Fig. 20c). For the soaking time of 12 h, N_2_ was close to the core’s outlet after the soaking time (Fig. 16e), and the shearing effect of the sand grains in the production period was weakened. It was easier to form the continuous gas phase. Therefore, the pressure gradient for the soaking time of 24 h was obviously higher than that for the soaking time of 12 h. It can be concluded that, increasing the soaking time was conducive to the migration of N_2_ to the top of formation, and the foamy oil flow was easier to form to improve the recovery performance.

## Conclusions

(1) The IFT between the crude oil and the N_2_ was reduced almost linearly with the pressure in two distinct pressure ranges. When the pressure was higher than 14.5 MPa, the trend of the IFT–pressure curve was affected by the accumulation of asphaltenes at the oil–N_2_ interface.

(2) The microscopic visual experiments indicated that there were significant differences among the behaviors of N_2_ flow during the three (1–3) cycles of N_2_ huff and puff process. In the first cycle, the N_2_ was dispersed in oil, forming the foamy oil flow. In the second cycle, the dispersed gas bubbles gradually coalesced into the continuous gas phase. In the third cycle, the foamy oil flow was not observed, while the N_2_ was produced in the form of continuous gas phase.

(3) The results from the tight oil coreflood experiments showed that, the primary recovery was only 5.32%, while the recoveries of the three (1–3) N_2_ huff and puff cycles were 15.1%, 8.53% and 3.22%, respectively. Because the foamy oil flow was formed in the first cycle, the recovery and pressure gradient in the first cycle were high, and the N_2_ production rate was low. With the increase in huff and puff cycles, the foamy oil flow weakened, while the oil recovery and the pressure gradient decreased rapidly.

## Electronic supplementary material

Supporting Information

Video Clip 1

Video Clip 2

Video Clip 3

Video Clip 4
