# Supplementary material for: Flow behavior of N2 huff and puff process for enhanced oil recovery in tight oil reservoirs
Source: Sci Rep. 2017 Nov 16;7:15695. doi: 10.1038/s41598-017-15913-5 (PMC5691204; doi:10.1038/s41598-017-15913-5)
Supplement: Supplementary file 1 — Supporting Information [file 41598_2017_15913_MOESM1_ESM.doc]

**Supporting Information**

**Flow behavior of N_2_ huff and puff process for enhanced oil recovery in tight oil reservoirs**

Teng Lu^a^*, Zhaomin Li^a^*, Jian Li^a^, Dawei Hou^a^ and Dingyong Zhang^b^

^a^School of Petroleum Engineering, China University of Petroleum, Qingdao 266580, China

^b^ Sinopec Shengli Oilfield Company, Dongying 257000, China

*Corresponding author’s e-mail: [luteng@upc.edu.cn](mailto:luteng@upc.edu.cn); [lizhm@upc.edu.cn](mailto:lizhm@upc.edu.cn)

List of Contents

S1. Video Clip 1 descriptions of primary production.

S2. Video Clip 2 descriptions of the first cycle of N_2_ huff and puff.

S3. Video Clip 3 descriptions of the second cycle of N_2_ huff and puff.

S4. Video Clip 4 descriptions of the third cycle of N_2_ huff and puff.
